# Adaptation and Validation of the Nursing Attitude Questionnaire in a Spanish Population

**DOI:** 10.3390/nursrep14040204

**Published:** 2024-10-03

**Authors:** Silvia Solera-Gómez, David Sancho-Cantus, Jesús Privado, Cristina Cunha-Pérez, Amparo Benedito-Monleón, Lucía Inmaculada Llinares-Insa

**Affiliations:** 1 Hospital Francisco de Borja, 46701, Valencia, Spain; solera_sil@gva.es; 2 Doctoral School, University of Valencia, 46600 Valencia, Spain; 3 Department of Nursing, Faculty of Medicine and Health Sciences, Catholic University of Valencia, 46600 Valencia, Spain; cristina.cunha@ucv.es; 4 Campus de Somosaguas, Department of Methodology of Behavioral Sciences, Universidad Complutense de Madrid, Pozuelo de Alarcón, 28223 Madrid, Spain; jesus.privado@pdi.ucm.es; 5 Faculty of Psychology, University of Valencia, 46000 Valencia, Spain; amparo.benedito@uv.es (A.B.-M.); lucia.llinares@uv.es (L.I.L.-I.)

**Keywords:** nursing, identity, stereotypes

## Abstract

Background: Nursing, as an essential component of contemporary healthcare systems, has undergone significant changes in recent decades, resulting in an increase in research across various fields, such as mental health, well-being, and the development of the discipline itself. Currently, gender stereotypes persist, impacting the social image of the profession and influencing the professional identity of nurses and consequently, their work performance and the quality of care provided. Both public and internal perceptions of the profession are critical factors in these dynamics. Objective: to adapt and validate the Nursing Attitude Questionnaire in a Spanish sample, as it has only been validated in two languages to date. Methods: This study presents descriptive and validity analyses of several measures utilized, such as Nursing Image (NIS), Medical Empathy (JSE-HP), Professional Values (NPVS) and Communication Skills (HP-CSS). Results: Confirmatory factor model analyses indicated that a 21-item hierarchical model provided a superior fit (χ^2^(df = 1.65, NFI = 0.954, SRMR = 0.065), suggesting the presence of a general factor accounting for five first-order factors. Internal consistency was deemed adequate for the total measures (α = 0.789), though variable for the subscales. Correlations among measures provided evidence of convergent and discriminant validity, highlighting consistent correlation patterns between Attitude toward Nursing and other variables. In addition, significant differences were observed in the Professionalism subscale according to sex, albeit with a medium-low effect size. Scales are presented to facilitate future interpretation of the results in similar samples. Conclusions: The scale validated in this study exhibited overall good psychometric performance, although certain limitations were identified within the subscales. The established scales represent a novel contribution, providing a valuable tool for the comparison of similar samples.

## 1. Introduction

### 1.1. Social Image of Nursing: Definition and Importance

The demand for highly qualified professionals to address the emerging health needs of the 21st century is increasingly growing. These needs must be patient-centered and serve as a reference model for the provision of quality care. Nursing is a fundamental component of health systems, contributing alongside other healthcare professionals to ensure the effective delivery of these services [1,2].

The nursing profession has undergone significant changes in recent decades [3] driven by factors such as increased life expectancy, the prevalence of chronic diseases, reduced hospitalization times, the technologization of health systems [4], and the evolving perception of patients as clients within these systems [5].

Since its emergence as a profession in the 19th century, nursing has been perceived as feminine and maternal, as caregiving was predominantly associated with women. Numerous studies have characterized nursing as a “profession constrained by an incoherent and outdated image, lacking leadership and subordinate to physicians”, prompting the American Nurses Association in 2021 to urge the nursing community to embrace effective and genuine leadership in society at large and within health systems specifically [6,7]. Florence Nightingale played a pivotal role in the professionalization of nursing, introducing values and beliefs related to the profession in her theories. Her key contributions included defining the distinct role of nurses, differentiating it from that of physicians, establishing a formal training model, and producing theoretical writings derived from practical actions [8].

Over time, and since its incorporation into the academic realm, nursing has evolved into a science characterized by rigorous university training that encompasses roles in management, research, and education, in addition to the caregiving functions it has historically performed. Gradually, nursing has moved away from its subordination to physicians, gaining autonomy in the execution of its functions [9]. The entry of men into nursing programs has also influenced shifts in the social image of the profession. Research has highlighted gender differences in the evaluation of the nursing profession [10,11]. Men tend to place greater emphasis on the technical aspects of nursing, while women generally express a stronger interest in relational dynamics, value patient care more highly, and tend to hold a more positive image of nursing [6,12]. Nevertheless, the persistence of gender stereotypes—such as the association of empathy and compassion with the female gender—contributes to nursing remaining a predominantly female profession (54,606 registered male nurses in 2023, compared to 291,363 registered female nurses) [13], perpetuating the associated social connotations [14].

The decision to pursue a career in nursing is influenced by numerous factors, including the historical context of the profession, media influence, familial connections to healthcare, and working conditions [15,16,17]. Among these, the image of the discipline itself is one of the most influential factors [18]. The professional identity (PI) of nurses encompasses the knowledge, skills, and societal beliefs surrounding nursing [19]. This perceived PI significantly impacts nurses’ job performance and their intention to remain in the profession [20]. When nurses perceive their social image as positive, they exhibit increased motivation and engagement in their work [21] and are often regarded as more competent [22]. A positive PI can also aid nurses in managing the demanding emotional requirements of their roles, reducing stress and burnout, and enhancing job satisfaction [23]. Key elements that constitute this PI include empathy, professional values, and communication skills. The significance of empathy lies in its effects on the patient–provider relationship: it enhances the quality of care, reduces stress, and improves clinical outcomes. Professional values guide clinical practice, ensuring quality and consistency in the patient and family care process and contributing to satisfaction and well-being within the healthcare environment. Furthermore, effective communication skills improve the efficiency of information gathering, facilitate the management of complex situations, and enhance patient satisfaction [19,20,21,23].

Moreover, the self-perception of nursing is influenced by various factors, including the quality of care provided [24], empathy, professional values, and communication skills. The initial exposure of nursing students to the clinical learning environment plays a crucial role in enhancing empathy levels and shaping their professional identity [25], which is further influenced by the professional values that guide nurses’ behavior [26]. Additionally, empathy is regarded by several authors as a fundamental communicative competence within professional practice [27].

Currently, it is recognized that both public opinion and the nurses themselves contribute to the development of nursing’s image in society [28,29]. Consequently, ongoing efforts are made to achieve greater recognition at the societal level [30]. Given that nurses derive their professional identity from society’s perception of them as well as their academic training, there exists a direct relationship between this identity, their job performance, and the quality of care provided [31,32]. Over the past decade, the perception of the professional prestige of nurses among healthcare professions has been inadequate, particularly within the community of healthcare professionals [33].

### 1.2. Measures of the Social Image of Nursing

There are various instruments used to understand society’s perception of nursing. Notable among them are:

- The Belgian Professional Self-Image Instrument for Nursing Students [34]: Designed to determine the issues affecting the recruitment and retention of nurses in Belgian hospitals.
- The Porter Nursing Scale [35]: Analyzes perception differences in nursing among nurses based on variables such as years of experience, type of shift, or training.
- The Professional Self-Concept of Nursing Instrument [36]: Evaluates constructs such as flexibility/creativity, knowledge, skill/competence, care, communication, leadership, and satisfaction.
- The Nurses Self-Concept Instrument [37]: Analyzes the self-concept of nurses both locally and internationally.
- The Nurses Self-Concept Questionnaire [38]: Designed to measure the self-concept of a group of nurses and to evaluate aspects related to nursing itself.
- The Nurse Self-Description Form [39]: Measures variables such as empathy, professionalism, and ethics in nursing work.
- The Nursing Brand Image Scale, Nursing Current Brand Position, and Nursing Desired Brand Position [40]: These three scales were designed to measure the corporate image of nursing.
- The Nursing Image Scale: Initially developed by Özsoy [41], the original version consisted of 35 items organized into five factors: general appearance, communication, working, education level, and profession-related suggestions.

### 1.3. Nursing Attitude Questionnaire (NAQ)

Designed by Toth et al. [42], the initial version comprises the following dimensions: nursing roles, values, professional activities, and responsibilities. This version consisted of 30 items rated on a Likert scale from 1 to 5, with higher scores indicating a more favorable perception of nursing. The content of the NAQ was derived from the earlier Hoskins Questionnaire [43], supplemented by a literature review, clinical experience, and recommendations from an expert panel of 11 members specializing in medical-surgical, psychiatric, and maternal-child nursing.

In the development and initial validation of the instrument, role theory, and socialization were employed as the theoretical framework, as the social role of nurses encompasses the beliefs, values, and behavioral patterns prevalent in society [44].

From this questionnaire, Toth designed the Nursing Image Questionnaire [44], an adapted version that intends to assess values and perceptions associated with the image of nurses as viewed by specific populations, such as the general public or nursing students. The questionnaire demonstrates internal consistency (Cronbach’s alpha) ranging from 0.75 to 0.80, with a test–retest reliability coefficient of 0.77 at four weeks. The internal discrimination of the items (i.e., the correlation of each item with the total score) reveals values exceeding 0.15. It consists of 30 Likert-type items, with response options ranging from strongly disagree to strongly agree. The questionnaire measures five dimensions: professional function (10 items), professional values (7 items), stereotypes (6 items), professional activities (4 items), and characteristics and responsibilities of nursing professionals (3 items).

An adaptation of the questionnaire to Croatian was conducted by Cukljek [44], which confirmed the five factors identified in previous versions and proposed a new version resulting from cultural adaptation. An exploratory principal components factor analysis was performed using varimax rotation and the Kaiser–Guttman criterion for retaining the principal factors. This analysis yielded a factorial structure with nine main components, accounting for 51.86% of the total variance of the manifest variables. Following rotation, non-interpretable factors emerged due to the presence of five factors: the role of nurses, values, social stereotypes concerning nursing, professionalism, and characteristics of nurses. Even after removing items with low saturation (<0.4), a fully interpretable factorial structure was not achieved across all factors, prompting the initiation of confirmatory factor analysis. The five retained factors explained 36% of the total variance, leading to the conclusion that the creation of a latent factor, as proposed by the original authors of the questionnaire, was not substantiated. The sample was predominantly female (86.3%), with 44.8% of respondents possessing up to 10 years of experience. The resulting internal consistency coefficient, measured by Cronbach’s alpha, was 0.63.

Validating and adapting this questionnaire for a Spanish nursing sample is essential, as this has not been previously undertaken. Furthermore, there exists only one adaptation of the questionnaire based on the original version, which does not yield satisfactory results or provide a comprehensive analysis of evidence concerning its internal structure. This adaptation is particularly significant due to the scarcity of validation studies for such instruments in the Spanish language, which are crucial for promoting their wider application.

The primary objective of this study was to adapt and validate the NAQ scale within a Spanish sample. This was achieved through the following specific objectives:

- Analyze the internal structure of the NAQ: Determine the number of dimensions that comprise it and assess whether these dimensions are hierarchically grouped under a general factor and associated specific factors, or if they are organized into correlated first-order factors.
- Calculate the internal consistency of each identified factor.
- Obtain evidence of convergent and discriminant validity by correlating the NAQ to other measures including nursing image, medical empathy, professional values, and communication skills.
- Investigate evidence of differential validity of the NAQ based on gender.
- Establish the norms for the evaluated sample.

## 2. Materials and Methods

### 2.1. Design

A cross-sectional design was utilized, with each participant evaluated only once.

### 2.2. Participants

The sample comprised 316 participants, with a mean age of 35.85 years (SD = 14.99), ranging from 17 to 78 years; 79.7% of the participants were women. In terms of educational attainment, 1.3% had completed primary education, 1.3% had achieved secondary education, 20.3% held a high school diploma, 24.4% had undergone vocational training, and 52.8% possessed a university degree.

### 2.3. Instruments

*Nursing Image Scale (NIS)* [37,45]. This scale assesses the public perception of the nursing profession through 35 items divided into the following dimensions: general impression (6 items), communication (6 items), working conditions (3 items), level of education (6 items), and profession-related suggestions (14 items). Responses are recorded on a three-point Likert scale, ranging from “agree” to “disagree”. The scale has been validated in a Spanish sample of 335 participants by Solera-Gómez et al. (in review), yielding a five-factor structure (GFI = 0.942, SRMR = 0.078) with adequate internal consistency (α = 0.84) for the overall scale. The internal consistency for the various tests administered in this study is presented in Table 1.

*Jefferson Scale of Empathy-Health Professionals Version* (JSE-HP) [46]. This instrument comprises 20 items rated on a Likert scale from 1 (strongly disagree) to 7 (strongly agree). It evaluates three dimensions: perspective-taking, compassionate care, and the ability to stand in the patient’s shoes. The version utilized in this study was specifically designed for health professionals.

*Nursing Professional Values Scale (NPVS)* [47]. This scale consists of 26 items that measure 11 dimensions of the American Nurses Association Code of Ethics, organized into five factors: care, activism, trust, professionalism, and justice. Responses are provided using a five-point analog Likert scale, ranging from 5 (very important) to 1 (not important).

*Health Professionals Communication Skills Scale (HP-CSS)* [48]. Comprising 18 items assessed on a six-point Likert scale (1 = almost never to 6 = very often), this scale encompasses four dimensions: informative communication, empathy, respect, and social skill.

*Nursing attitude questionnaire (NAQ)* (Appendix A) [38]. This questionnaire consists of 30 items that evaluate stereotypes, values, professionalism, roles, and characteristics of nursing.

### 2.4. Procedure

The adaptation process included several steps: bidirectional translation, collaborative discussion, testing a draft version of the questionnaire with a small group, and the development of the final version (Appendix B). Initially, two independent translators rendered the original text into Spanish. After reaching a consensus, the translated version was translated back into English by two different experts in translation [49]. The two versions were reconciled, resulting in a final version that received approval from the original author. The questionnaire was administered via social media using the “snowball sampling” technique [50] to maximize participant recruitment. This method is inherently subject to bias, as the sample is not randomly selected from the population. Participants were informed that their involvement in the study was voluntary and anonymous, and that no financial compensation would be provided. They received information regarding the study’s general aspects prior to completing the questionnaire. Given the purposive sampling approach, outreach efforts were made to various healthcare centers, nursing colleges, associations, and universities to facilitate questionnaire dissemination. All participants provided signed informed consent. The inclusion criteria encompassed individuals aged over 18 years who demonstrated adequate proficiency in the Spanish language and who did not have any cognitive limitations that could hinder their comprehension of the questionnaire.

### 2.5. Ethical Considerations

The study was conducted in accordance with the principles outlined in the *Declaration of Helsinki* [51] and received approval from the Human Research Committee of the University of Valencia (procedure number 2023-ENFPOD-2638296). Participants included in the study provided informed consent after being thoroughly informed about the procedures and the nature of the study.

### 2.6. Data Analysis

First, the distribution of the various measures employed, and their internal consistency were assessed using Cronbach’s alpha. Second, the factorial structure of the Nursing Attitude Questionnaire (NAQ) was analyzed through confirmatory factor analysis (CFA). To evaluate the fit of the data, three types of goodness-of-fit indices were utilized: Absolute Fit Indices, which was used to assess whether the theoretical model aligned with the empirical data, including the χ^2^/df index [52] (with values below 3 indicating a good fit), the Goodness-of-Fit Index (GFI) [53] (with values greater than 0.95 indicating a good fit), and the Standardized Root Mean Square Residual (SRMR) [54] (with values less than 0.08 indicating a good fit). The Incremental Fit Indices, which compared the obtained model to a null model were also employed; specifically, the Normed Fit Index (NFI) [55] (with values greater than 0.95 indicating a good fit). Additionally, the Parsimonious Fit Indices, which penalize the number of estimated parameters, included the Parsimony Goodness-of-Fit Index (PGFI) [53] and the Parsimony Normed Fit Index (PNFI) [56], both with values greater than 0.50 indicating a good fit.

It is recommended to have 10 participants per indicator for factor analyses [57]; in this study, there were 316 participants and 30 indicators in the most complex model, resulting in a ratio of 316/30 = 10.53, indicating an adequate sample size for the model. The estimated power, calculated based on the sample size, a significance level of 0.05, and a regression weight of 0.02, was found to be 0.81. According to Cohen’s (1992) criteria for linear regression, an effect size of f2 = R2(1−R2)f2 = (1−R2)R2 of 0.02 is classified as a low effect size, 0.15 as a medium effect size, and 0.35 as a high effect size. For power estimation purposes, the most conservative effect size of 0.02 was employed.

Third, the internal consistency of the total test and its dimensions was calculated, along with the correlation of each item with the corrected total dimension to evaluate the internal discrimination of each dimension. Fourth, Pearson correlations of the scale and its dimensions with two additional scales and their dimensions were computed to provide evidence of convergent and discriminant validity. A confirmatory model was also applied, correlating the different factors from the administered questionnaires to observe the correlation patterns. Fifth, the evidence of differential validity of the test based on gender was examined using a Student’s *t*-test. Lastly, the norms of the test were calculated, including percentile scores, Z scores, normalized Z scores, and T scores.

All analyses were conducted using SPSS V.23 and AMOS V.23 statistical packages [58].

## 3. Results

### 3.1. Descriptive Analysis

Table 2 presents the descriptive statistics for the various measures utilized in this study. With the exception of the Ethics dimension of the Nursing Professional Values Scale (NPVS) and one measure from the Nursing Attitude Questionnaire (NAQ) related to Professionalism, the skewness of the data does not exceed |±2|, nor does the kurtosis exceed |±7|, indicating that most measures exhibit a normal distribution [59]. Furthermore, the internal consistency, as assessed by Cronbach’s alpha, for the measures of Nursing Image (NIS), Medical Empathy (JSE-HP), Professional Values (NPVS), and Communication Skills (HP-CSS) is generally adequate, with values of at least 0.70 [55]. However, the subscales for NIS, Empathy (JSE-HP), and Social Skills (HP-CSS) do not meet this threshold. Nevertheless, the total scores for these measures—Nursing Image (NIS), Medical Empathy (JSE-HP), and Communication Skills (HP-CSS)—demonstrate adequate internal consistency (α > 0.70). Therefore, results from these subscales should be interpreted with caution.

### 3.2. Internal Validity Evidence

A confirmatory model comprising five correlated factors, as well as a hierarchical model featuring a second-order factor elucidating the five first-order factors, was estimated. The models did not demonstrate multivariate normality, as indicated by the Bollen-Stine bootstrap test [59] (*p* = 0.005); therefore, they were estimated using unweighted least squares, which do not necessitate this assumption. Table 3 presents the goodness-of-fit indices for the various models tested. The five-factor model with 30 items exhibited a moderate fit to the data; however, some items displayed very low factor loadings (<0.40), which is below the minimum threshold recommended by Hair et al. [55]. Consequently, items with loadings below 0.40 were removed, and the model was re-estimated, resulting in improved fit and adequate factor loadings.

**Figure 1:**
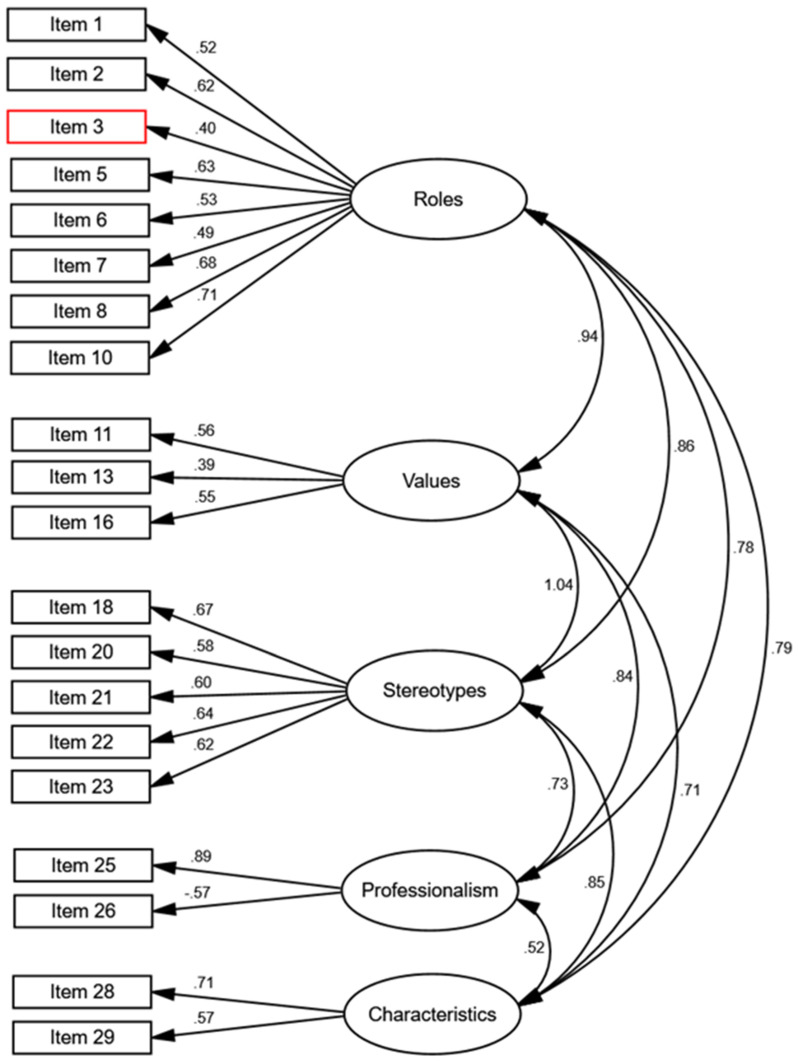
Model of Five Correlated Factors for NAQ.

**Figure 2:**
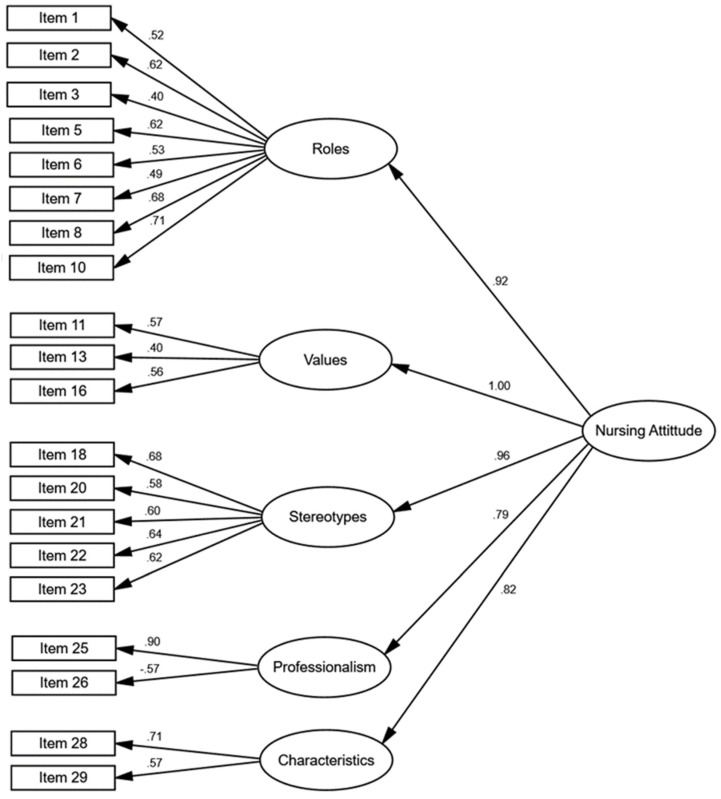
Hierarchical Model for NAQ.

Figure 1 illustrates the five factors along with the final items constituting each factor. Certain factors, such as roles and stereotypes, include a greater number of items, while others are characterized by fewer items. Nevertheless, all factors displayed adequate factor loadings (>|±0.39|). The revised 21-item model revealed high correlations among factors, many exceeding 0.80, suggesting the presence of a second-order factor that explains the five first-order factors. Subsequently, a hierarchical confirmatory model using the original 30 items was estimated, which also exhibited a good fit, although it contained nine items with factor loadings <0.40. Upon removing these items, the hierarchical model was re-estimated with 21 items (see Figure 2), demonstrating superior fit compared to the 30-item model and featuring general factor loadings for the five first-order factors ranging from 0.79 to 1.00, thereby justifying the existence of this general factor. While both the five-factor and hierarchical 21-item models displayed a similar fit to the data (refer to Table 3), the hierarchical model is deemed more coherent due to the high correlations among the five first-order factors (>0.80) [55]. Consequently, the factorial structure of the scale is characterized by a general second-order factor (Nursing Attitude) encompassing five first-order factors (Roles, Values, Stereotypes, Professionalism, and Characteristics), collectively accounting for 21 items within the questionnaire. The most prominent first-order factors, Values and Stereotypes, exhibited higher factor loadings (1.00 and 0.96, respectively). In contrast, the factors of Characteristics and Professionalism were less significant in elucidating the measure, with lower factor loadings (0.82 and 0.79, respectively). Overall, all five factors significantly contribute to the explanation of the items within the questionnaire.

### 3.3. Reliability Evidence of the 21-Item Test

Internal consistency for the Nursing Attitude Questionnaire (NAQ) was evaluated using Cronbach’s alpha for both the subscales and the overall test. The results, presented in Table 2, indicate acceptable internal consistency for the total test (α = 0.789); however, this threshold was not met by all subscales. The minimum recommended value is 0.70 [55], and none of the five subscales exceeded this minimum, with the highest subscale score being 0.774. Consequently, results at the subscale level should be interpreted with caution. The internal discrimination of each subscale was assessed by correlating each item with the corrected total score of the respective scale. Most items surpassed the minimum correlation value of 0.20 [55], except for two items within the Values subscale. Despite this, the Values subscale exhibited a factor loading of 1.00 with the general factor in the internal structure, leading to the decision not to exclude it from the model. Furthermore, the correlation of each item with the total test was calculated to evaluate the internal discrimination of the overall test, with only item 26 exhibiting a correlation value below 0.20 (specifically 0.18). Thus, although the internal discrimination of the Values subscale is low, the overall test demonstrates adequate internal discrimination.

### 3.4. Convergent and Discriminant Validity Evidence

**Figure 3:**
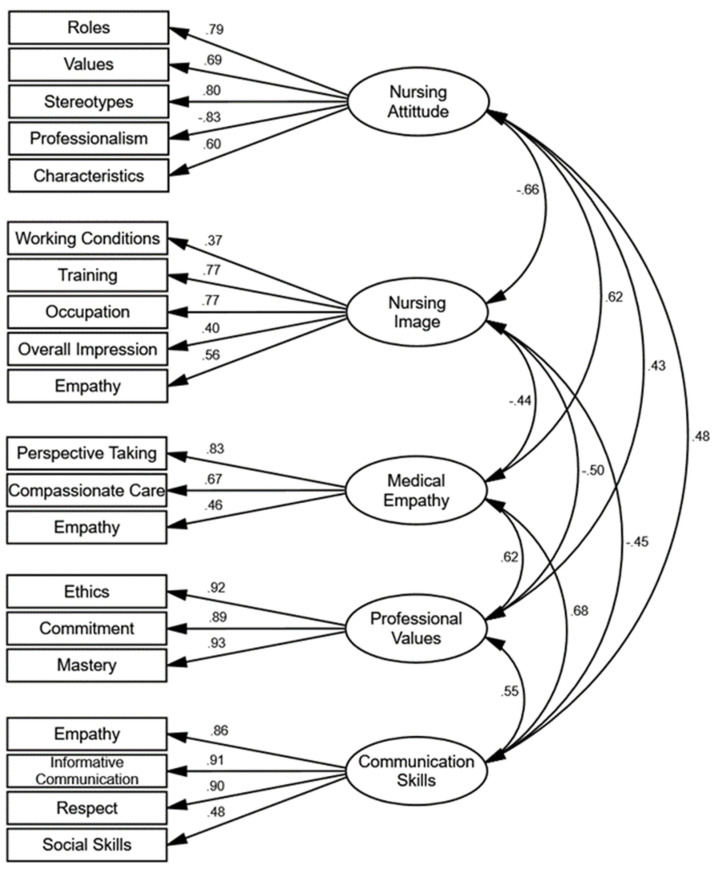
Convergent Validity Model.

Pearson correlations were calculated between the various subscales and the total score of the Nursing Attitude Questionnaire (NAQ), as well as with other scales and subscales (Nursing Image Scale [NIS], Jefferson Scale of Empathy-Health Professionals Version [JSE-HP], Nursing Professional Values Scale [NPVS], and Health Professionals Communication Skills Scale [HP-CSS]) (see Table 4). Employing a minimum correlation threshold of |±0.30|, which denotes a medium effect size according to Cohen [60], it is evident that the NAQ demonstrates numerous correlations exceeding this threshold with the different measures. Specifically, the subscales of Roles, Values, Stereotypes, Characteristics, and the overall NAQ score exhibit negative correlations with the NIS while showing positive correlations with the other measures. Conversely, the Professionalism subscale correlates positively with all measures. Additionally, a confirmatory model was estimated that correlates the factors derived from the NAQ with those from the other measures. Figure 3 illustrates this tested model, and Table 3 presents the goodness-of-fit indices, which indicate a good fit for the data. Notably, Nursing Attitude shows a negative correlation with Nursing Image (β = −0.66) and positive correlations with Medical Empathy (β = 0.62), Professional Values (β = 0.43), and Communication Skills (β = 0.48). Consequently, the Nursing Attitude scale exhibits medium-high convergent correlation patterns with other measures pertinent to its constructs.

### 3.5. Differential Validity Evidence

Mean differences were assessed using a Student’s *t*-test for the NAQ subscales and the total score. The results, presented in Table 5, reveal statistically significant differences solely for the Professionalism subscale, with men scoring higher. Effect size was calculated using Cohen’s d [60], where values of 0.20 are classified as small, 0.50 as medium, and 0.80 as large. The observed effect sizes for mean differences range from 0.05 to 0.36, indicating medium-low effects. These findings suggest minimal gender differences within the NAQ.

### 3.6. Norms/Scales

Appendix C presents the norms for the study participants. For each raw score, the percentile, standardized (Z), normalized Z, and T scores (M = 50, SD = 10) were calculated. These data will facilitate future evaluators in assessing the position of their participants relative to a comparable sample. Norms were computed for all participants without gender differentiation, as the only scale exhibiting differences was Professionalism, which demonstrated a medium-low effect size (d = 0.36).

## 4. Discussion

In this study, the NAQ scale was adapted and validated with a sample of 316 participants from various educational backgrounds. This scale was selected due to its status as one of the few tools available for measuring attitudes toward nursing, and it has undergone only two previous validations in different contexts, thereby enhancing its relevance [61,62].

The findings indicate that the internal structure of the NAQ aligns with both a model consisting of five first-order correlated factors—Roles, Values, Stereotypes, Professionalism, and Characteristics—as originally proposed by the authors [38]. Additionally, a hierarchical model, wherein a general second-order factor elucidates the five first-order factors, is supported. Given the high correlations among the first-order factors (many exceeding 0.80), a hierarchical internal structure is deemed more appropriate. In this model, 21 of the original 30 NAQ items were retained, as nine items exhibited factor loadings below 0.40. The internal consistency for the overall scale was adequate (α = 0.789); however, the subscale internal consistencies were not. Consequently, we recommend utilizing the total scale for future evaluations. This aligns with Toth’s findings (α = 0.75–0.80) and represents an improvement over Cukljek’s results (α = 0.63) [40].

Concerning the evidence for convergent and discriminant validity of the NAQ, the test demonstrated negative correlations with Nursing Image. The original authors indicated that nursing is perceived as a passionate and significant profession, which contrasts with our findings. One possible explanation for these discrepancies could be the timing of the study, conducted after the COVID-19 pandemic, which likely altered public perceptions of the nursing profession [63]. During the pandemic, the social image of nursing improved substantially, primarily due to increased public visibility and recognition of nurses’ roles.

The analyses further revealed a positive relationship between the NAQ and other measures, including Medical Empathy, Professional Values, and Communication Skills, consistent with existing literature. However, when examining results by gender, significant differences emerged in the value attributed by women to ethical principles and professional commitment [64], potentially linked to the traditionally assigned caregiving roles associated with females. Gender differences in empathy were also noted, with women scoring higher in both cognitive and emotional empathy. Furthermore, students exhibiting elevated levels of empathy demonstrated superior communication skills and a heightened valuation of their profession [65,66].

Moreover, minimal gender differences were observed across the various measures of the test; notably, only males exhibited higher scores in the Professionalism subscale, with a medium-low effect size (d = 0.36).

### 4.1. Implications of the Present Study

Understanding the social image of nursing has significant implications across multiple dimensions, including the alignment of expectations regarding the profession and the enhancement of nursing curricula.

### 4.2. Limitations

The principal limitation of this study relates to the sample size and the participant selection method. Therefore, future research should aim to enhance the sample size and mitigate selection biases. Additionally, achieving an equitable distribution of participants by sex, considering diverse age groups, and comparing results among them was not feasible, nor was it possible to account for participants’ professional experience based on years of service in patient care within the healthcare sector. Future studies should prioritize a more targeted sample selection to control for specific demographic groups.

A mixed-methods study design could be proposed, allowing qualitative approaches to provide deeper insights into the social representation of the nursing profession. Furthermore, participant feedback would be invaluable in refining the findings related to the relevance of the items concerning the scale’s objectives, thus complementing the psychometric analysis conducted. A longitudinal study could also be beneficial to examine the temporal stability of the scale.

## 5. Conclusions

The validated scale has demonstrated robust psychometric performance, although certain challenges remain concerning some subscales. The existence of five primary factors, as identified in prior validations of the instrument has been reaffirmed alongside the minimal gender-based differences in perceptions of the nursing profession. The normative data established in this study were previously unavailable, thus offering a valuable resource for comparisons with similar samples.

The direct implications of this study for the nursing field relate to the potential for a deeper understanding of both the projected and perceived images of the discipline. In this context, it is crucial for nursing professionals to engage in reflective practices regarding their achievements and current positioning. Such reflection will enable them to accurately articulate their professional identity and goals, as well as communicate these effectively to society.

## Figures and Tables

**Table 1:** Instruments measuring the social image of Nursing.

Instrument	Author/s	Sample	Factorial Structure	Reliability	Validity
Porter Nursing Scale	Porter and Porter (1991)	303 nurses (90% women)	3: interpersonal power, interpersonal skills, interpersonal relationships	α = 0.85	
The Nursing Attitudes/Image Questionnaire (NAQ/NIQ)	Toth et al. (1998)	338 people. Six of the traditional students were enrolled in a Bachelor of Science in nursing degree/Master of Science in nursing degree program, 31 were RNs, 22 held associate’s degrees, and 9 were diploma graduates.	5 dimensions, 30 items: 1. Roles 2. Values 3. Stereotypes 4. Professionalism 5. Nursing characteristics	α = 0.75–0.80	Content Validity: Determined by a panel of undergraduate nursing students. Construct Validity: Measured by comparing 45 registered nurses with 45 non-registered nurses.
Belgian professional self-image instrument for nursing students,	Siebens et al. (2006)	9638 nurses from 22 hospitals in Belgium	3 dimensions, 52 items: 1. Competence 2. Nursing care 3. Team		
The Professional Self-concept of Nurses Instrument	Arthur (1995)	170 third-year nursing students	3 dimensions, 5 subdimensions, 27 items: 1. Professional practice 2. Satisfaction 3. Communication	α = 0.89	EFA: 40%–57.30% of the variance was explained (n = 170)
The Nurses Self-Concept Instrument	Angel et al. (2012)	253 university nursing students from Sydney	4 dimensions, 14 items: 1. Care 2. Knowledge 3. Relationship with the staff 4. Leadership	α = 0.94	CFA showed good fit of the original factor structure, RMSEA = 0.066 (95% CI: 0.051–0.080), NNFI = 0.98, CFI = 0.99, x^2^ = 148.89, *df* = 71, *p*-value for test of close fit = 0.025
The Nurses Self-Concept Questionnaire, NSCQ	Cowin (2001)	506 undergraduate nursing students and 508 practicing nurses	6 dimensions, 36 items: 1. General nursing 2. Care 3. Relationship with the staff 4. Communication 5. Knowledge 6. Leadership	α = 0.90	CFA: 72.9% of the variance was explained. CFA showed good fit, TLI = 0.911, RNI = 0.887, GFI = 0.906, x^2^ = 787.05, *df* = 120
The Nurse Self-Description Form, NSDF	Dagenais and Meleis (1982)	186 graduate nurses	3 dimensions, 19 items: 1. Professionalism 2. Empathy 3. Ethical work	α = 0.90	EFA: 62.5 of the variance was explained
The Nursing Brand Image Scale, NBIS	Godsey et al. (2018)	286 participants, 152 nurses	7 dimensions, 42 sentences: 1. Leaders’ influence 2. Qualification for advanced nursing practice 3.Qualified caregivers 4. Strong interpersonal skills 5. Lack of authority 6. Expert healthcare providers 7. Appreciation by society	α = 0.92	EFA: 61.6% of the variance was explained
Nursing’s Current Brand Position Scale, NCPBS	Godsey et al. (2018)	286 participants, 152 nurses	3 dimensions, 10 items: 1. Influence of leaders 2. Patient-centered care 3. Patient advocates	α = 0.92	CFA: 68.4% of the variance was explained
Nursing’s Desired Brand Position Scale, NDBPS	Godsey et al., 2018	286 participants, 152 nurses	2 dimensions, 10 items: 1. Influence of leaders 2. Patient-centered care	α = 0.90	CFA: 68.3% of the variance was explained
Nurse Image questionnaire	Özsoy, 2000		5 factors: General Impression, Communication, Working Conditions, Level of Education, and Suggestions Related to the Profession	The internal consistency coefficient is α = 0.709	The scale was validated on a sample of 500 individuals in Turkey, with a gender distribution of 50.0% women. Of the total sample, 44.4% had completed secondary education

Note. EFA = exploratory factor analysis; CFA = confirmatory factor analysis; RMSEA = Root Mean Square Error of Approximation; NNFI = Non-Normed Fit Index; CFI = Comparative Fit Index; CI = confidence interval.

**Table 2:** Descriptive Statistics, Internal Consistency (Cronbach’s α), and Internal Discrimination (Item-Total Corrected Correlation) for the Measures.

Measures	M	SD	Skewness	Kurtosis	α	Item-Total Corrected Correlation
Roles (NAQ)	33.48	3.84	−0.57	1.30	0.652	0.19 to 0.57
Values (NAQ)	12.96	1.70	−0.42	−0.58	0.125	0.05 to 0.09
Stereotypes (NAQ)	21.28	2.76	−0.53	−0.34	0.614	0.26 to 0.45
Professionalism (NAQ)	2.60	1.18	2.73	8.87	0.493	0.33
Characteristics (NAQ)	8.10	1.46	−0.63	−0.04	0.491	0.33
Nursing attitude (NAQ)	78.42	7.02	−0.25	−0.55	0.789	0.18 to 0.53
Working Conditions (NIS)	4.17	1.15	0.71	−0.36	0.394	
Education (NIS)	6.91	1.46	2.00	4.26	0.626	
Occupation (NIS)	11.50	2.14	0.88	0.70	0.579	
General View (NIS)	5.75	1.49	0.59	−0.67	0.664	
Empathy (NIS)	8.62	2.33	0.69	−0.51	0.765	
Nursing Image (NIS)	36.95	5.98	0.93	1.18	0.826	
Perspective-Taking (JSE-HP)	62.65	6.93	−1.17	1.07	0.820	
Compassionate Care (JSE-HP)	41.52	7.21	−1.13	1.09	0.713	
Empathy (JSE-HP)	14.22	3.13	−0.37	0.23	0.343	
Medical Empathy (JSE-HP)	118.39	13.86	−0.89	0.48	0.832	
Ethics (NPVS)	42.44	3.80	−2.12	4.93	0.845	
Commitment (NPVS)	36.90	3.76	−1.48	1.81	0.863	
Mastery (NPVS)	41.62	4.10	−1.65	2.66	0.840	
Professional Values (NPVS)	120.95	10.92	−1.91	3.91	0.941	
Empathy (HP-CSS)	26.82	3.65	−1.74	3.97	0.893	
Informative Communication (HP-CSS)	31.29	4.01	−1.24	2.36	0.801	
Respect (HP-CSS)	16.36	2.05	−1.69	3.99	0.832	
Social Skills (HP-CSS)	17.90	3.24	−0.06	-0.33	0.561	
Total Communication Skills (HP-CSS)	97.68	11.37	−1.27	2.75	0.913	

Note: M = mean; SD = standard deviation; α, Cronbach’s alpha.

**Table 3:** Goodness-of-fit indices for the models.

Model	χ^2^*/df*	GFI	NFI	PGFI	PNFI	SRMR
Five correlated factors with 30 items	3.55	0.907	0.836	0.770	0.750	0.078
Five correlated factors with 21 items	1.62	0.972	0.956	0.740	0.805	0.063
Hierarchical model with 30 items	3.52	0.906	0.825	0.782	0.761	0.078
Hierarchical model with 21 items	1.65	0.970	0.954	0.767	0.833	0.065
Convergent Validity	143.15	0.995	0.990	0.758	0.834	0.055

Note: χ^2^/*df* = chi square; GFI = Goodness-of-Fit Index; NFI = Normed Fit Index; PGFI = PaRSIMONY Goodness-of-Fit Index; PNFI = Parsimony Normed Fit Index; SRMR = Standardized Root Mean Square Residual.

**Table 4:** Pearson correlations between NAQ, NIS, JSE-HP, EVPE, and EHC-PS.

	Roles (NAQ)	Values (NAQ)	Stereotypes (NAQ)	Professionalism (NAQ)	Characteristics (NAQ)	Nursing Attitude (NAQ)
Working Conditions (NIS)	−0.16	−0.21	−0.15	0.21	−0.09	−0.17
Education (NIS)	**−0.31**	**−0.35**	**−0.32**	**0.34**	−0.17	**−0.33**
Occupation (NIS)	**−0.34**	**−0.43**	**−0.41**	**0.31**	−0.26	**−0.42**
General View (NIS)	−0.26	−0.20	−0.17	0.16	−0.21	−0.25
Empathy (NIS)	**−0.41**	−0.26	−0.29	0.18	−0.29	**−0.40**
Nursing Image (NIS)	**0.45**	**0.42**	**0.40**	**0.34**	**0.31**	**0.48**
Perspective-Taking (JSE-HP)	**0.42**	**0.34**	**0.42**	**0.49**	**0.32**	**0.43**
Compassionate Care (JSE-HP)	**0.31**	0.27	0.27	**0.45**	0.20	0.28
Empathy (JSE-HP)	0.11	0.12	0.13	0.19	0.07	0.11
Medical Empathy (JSE-HP)	**0.42**	**0.37**	**0.41**	**0.53**	**0.31**	**0.42**
Ethics (NPVS)	**0.30**	0.27	**0.35**	**0.31**	0.21	**0.33**
Commitment (NPVS)	0.29	**0.31**	**0.34**	**0.30**	0.19	**0.33**
Mastery (NPVS)	**0.30**	**0.30**	**0.32**	**0.36**	0.19	**0.32**
Professional Values (NPVS)	**0.31**	**0.31**	**0.35**	**0.34**	0.21	**0.35**
Empathy (HP-CSS)	**0.39**	0.29	**0.42**	0.28	**0.32**	**0.43**
Informative Communication (HP-CSS)	**0.36**	**0.30**	**0.39**	**0.31**	**0.33**	**0.40**
Respect (HP-CSS)	**0.40**	**0.31**	**0.45**	**0.32**	**0.35**	**0.45**
Social Skills (HP-CSS)	0.21	0.16	0.24	0.15	0.15	0.24
Total Communication Skills (HP-CSS)	**0.40**	**0.31**	**0.44**	**0.31**	**0.34**	**0.45**

Note. Correlations ≥|±0.11| are statistically significant at the 5% level. Correlations ≥|±0.30| are in bold.

**Table 5:** Descriptive Statistics, Mean Differences, and Effect Size for the NAQ.

Measures	Male M (SD)	Female M (SD)	*t*-Test	Cohen’s d
Roles	33.91 (4.68)	33.38 (3.61)	*t*_314_ = 0.98, *p* = 0.326	0.14
Values	12.81 (1.79)	13.00 (1.68)	*t*_314_ = −0.79, *p* = 0.431	0.11
Stereotypes	21.17 (2.81)	21.31 (2.75)	*t*_314_ = −0.36, *p* = 0.722	0.05
Professionalism	2.94 (1.57)	2.51 (1.05)	*t*_78_ = 2.59, *p* = 0.044	0.36
Characteristics	7.92 (1.57)	8.14 (1.43)	*t*_314_ = -1.08, *p* = 0.280	0.15
Nursing Attitude	78.75 (7.48)	78.34 (6.91)	*t*_314_ = 0.42, *p* = 0.678	0.06

Note: M, mean; SD, Standard deviation; d, effect size.

## Data Availability

The data presented in this study are available on request from the corresponding author.

## Public Involvement Statement

No public involvement in any aspect of this research.

## Guidelines and Standards Statement

This study was performed in accordance with the Strengthening the Reporting of Observational Studies in Epidemiology (STROBE) Checklist (Cross-sectional studies), consists of an analysis of the psychometric properties of a questionnaire, using reference materials in the field, such as that of Abad et al. (Abad F.J.; Olea, J.; Ponsoda, V.; García, G. Medición en ciencias sociales y de la salud, Ed. Síntesis, Spain. 2014).

## Appendix A. *Nursing Attitude Questionnaire (Original Version)*

1. Nurses are patient advocates.
2. Nurses protect patients in the healthcare system.
3. Nursing professionals participate in the development of healthcare policy.
4. Nursing professionals must wear white uniforms in order to be recognized.
5. Nursing professionals are a resource for people with health problems.
6. Nursing professionals are, in general, compassionate and kind people.
7. It takes intelligence to be a nurse.
8. The service provided by nurses is as important as the service provided by physicians.
9. Everyone would benefit if nurses spent fewer hours in training and more time caring for patients.
10. Nurses integrate theory and practice.
11. Research is a vital part of the nursing profession.
12. Nursing professionals are active in the political arena.
13. Nursing professionals are able to work independently.
14. Nurses speak out against undignified working conditions.
15. The certainty that they are fasting people at work is sufficient compensation for nurses.
16. Nurses should have the right to strike.
17. Nursing professionals follow doctor’s orders without questioning them.
18. Men make good nurses.
19. Many of the nursing professionals would actually prefer to be physicians.
20. Nursing is exciting.
21. Nursing professionals include research findings in their clinical practice.
22. The main objective of nursing research is to improve patient care.
23. Nursing professionals value the time spent at the bedside caring for their patients.
24. Nursing professionals should have a university degree for internships.
25. Nurses make important contributions to patient care.
26. One advantage of being a nurse is being able to marry a physician.
27. Nursing is a respected profession.
28. Nurses feel good about what they do.
29. Nursing practice is continually being updated in relation to current health trends.
30. Nurses are paid an adequate salary for the work they perform.

## Appendix B. *Nursing Attitude Questionnaire (Spanish Version)*

1. Los profesionales de enfermería son defensores de los pacientes.
2. Los profesionales de enfermería protegen a los pacientes en el sistema de salud.
3. Los profesionales de enfermería participan en el desarrollo de políticas de salud.
4. Los profesionales de enfermería deben llevar uniforme blanco para poder ser reconocidos.
5. Los profesionales de enfermería son un recurso para las personas con problemas de salud.
6. Los profesionales de enfermería son, en general, personas compasivas y amables
7. Se requiere inteligencia para ser enfermera.
8. El servicio prestado por los profesionales de enfermería es tan importante como el que prestan los médicos.
9. Todo el mundo se beneficiaría si los profesionales de enfermería pasaran menos horas formándose y más tiempo cuidando a pacientes.
10. Los profesionales de enfermería integran teoría y práctica.
11. La investigación es parte vital de la profesión enfermera.
12. Los profesionales de enfermería son activos en el ámbito político.
13. Los profesionales de enfermería son capaces de trabajar de forma independiente.
14. Los profesionales de enfermería alzan la voz contra las condiciones laborales indignas.
15. La certeza de que están ayunando a gente en su trabajo es compensación suficiente para los profesionales de enfermería.
16. Los profesionales de enfermería deberían tener derecho a la huelga.
17. Los profesionales de enfermería siguen las órdenes del médico sin cuestionarlas.
18. Los hombres son buenos profesionales de enfermería.
19. Muchos de los profesionales de enfermería en realidad preferirían ser médicos.
20. La enfermería es emocionante.
21. Los profesionales de enfermería incluyen en su práctica clínica resultados de investigación.
22. El objetivo principal de la investigación en enfermería es mejorar la atención al paciente.
23. Los profesionales de enfermería valoran el tiempo a pie de cama cuidando a sus pacientes.
24. Los profesionales de enfermería deberían tener el título universitario para hacer prácticas.
25. Los profesionales de enfermería hacen contribuciones importantes al cuidado de los pacientes.
26. Una ventaja de ser enfermera es poder casarse con un médico.
27. La enfermería es una profesión respetada.
28. Las enfermeras se sienten bien con lo que hacen.
29. La práctica enfermera está continuamente actualizándose en relación a las tendencias de salud actuales.
30. Los profesionales de enfermería reciben un sueldo adecuado por el trabajo que realizan.

## Appendix C. Norms for NAQ

**Percentile**	**Z_n_**	**T**	**Roles**	**Z_Roles_**	**Values**	**Z_Values_**	**Stereotypes**	**Z_Stereotypes_**	**Professionalism**	**Z_Professionalism_**	**Characteristics**	**Z_Characteristics_**	**Total**	**Z_Total_**
1	−2.33	26.70	24.17	−2.42	8.17	−2.82	13.17	−2.94	2.00	−0.51	4.00	−2.81	62.17	−2.31
5	−1.64	33.60	27.85	−1.47	10.00	−1.74	17.00	−1.55	2.00	−0.51	5.00	−2.12	66.00	−1.77
10	−1.28	37.20	29.00	−1.17	11.00	−1.15	17.70	−1.30	2.00	−0.51	6.00	−1.44	68.00	−1.48
15	−1.04	39.60	30.00	−0.91	11.00	−1.15	18.00	−1.19	2.00	−0.51	6.00	−1.44	70.00	−1.20
20	−0.84	41.60	30.00	−0.91	11.00	−1.15	19.00	−0.83	2.00	−0.51	7.00	−0.75	72.00	−0.91
25	−0.67	43.30	31.00	−0.65	12.00	−0.56	19.00	−0.83	2.00	−0.51	7.00	−0.75	74.00	−0.63
30	−0.52	44.80	31.10	−0.62	12.00	−0.56	20.00	−0.46	2.00	−0.51	8.00	−0.07	75.00	−0.49
35	−0.39	46.10	32.00	−0.39	12.00	−0.56	20.00	−0.46	2.00	−0.51	8.00	−0.07	76.00	−0.34
40	−0.25	47.50	33.00	−0.12	12.80	−0.09	21.00	−0.10	2.00	−0.51	8.00	−0.07	77.80	−0.09
45	−0.13	48.70	33.00	−0.12	13.00	0.02	21.00	−0.10	2.00	−0.51	8.00	−0.07	78.00	−0.06
50	0.00	50.00	34.00	0.14	13.00	0.02	22.00	0.26	2.00	−0.51	8.00	−0.07	79.00	0.08
55	0.13	51.30	34.00	0.14	13.00	0.02	22.00	0.26	2.00	−0.51	8.00	−0.07	80.00	0.23
60	0.25	52.50	35.00	0.40	14.00	0.61	22.00	0.26	2.00	−0.51	9.00	0.62	81.00	0.37
65	0.39	53.90	35.00	0.40	14.00	0.61	23.00	0.62	2.00	−0.51	9.00	0.62	82.00	0.51
70	0.52	55.20	36.00	0.66	14.00	0.61	23.00	0.62	3.00	0.34	9.00	0.62	82.00	0.51
75	0.67	56.70	36.00	0.66	15.00	1.20	23.00	0.62	3.00	0.34	9.00	0.62	83.00	0.65
80	0.84	58.40	37.00	0.92	15.00	1.20	24.00	0.99	3.00	0.34	9.00	0.62	84.60	0.88
85	1.04	60.40	38.00	1.18	15.00	1.20	24.00	0.99	3.00	0.34	10.00	1.30	86.00	1.08
90	1.28	62.80	38.00	1.18	15.00	1.20	25.00	1.35	4.00	1.19	10.00	1.30	88.00	1.36
95	1.64	66.40	40.00	1.70	15.00	1.20	25.00	1.35	6.00	2.88	10.00	1.30	89.00	1.51
99	2.33	73.30	40.00	1.70	15.00	1.20	25.00	1.35	7.83	4.43	10.00	1.30	92.00	1.93
n	316	316	316	316	316	316	316	316	316	316	316	316	316	316
Mean	0.00	50.00	33.48	0.00	12.96	0.00	21.28	0.00	2.60	0.00	8.10	0.00	78.42	0.00
SD	1.00	10.00	3.84	1.00	1.70	1.00	2.76	1.00	1.18	1.00	1.46	1.00	7.02	1.00
Note: n, sampling size; T, T-score; Z, standardized score; Zn, normalized standard score.														

## References

[1] Braš M., Đordević V., Pjevač N., Kaštelan S., Klarica M., Orešković S. (2022). How to teach person-centered medicine during the coronavirus disease 2019 pandemic?. Croat Med. J..

[2] Salihović A., Mahmutović J., Branković S. (2024). Nursing students’ attitudes about their profession. J. Health Sci..

[3] Wakefeld M., Williams D.R., Le Menestrel S. (2021). The Future of Nursing 2020–2030: Charting a Path to Achieve Health Equity.

[4] Archibald M.M., Barnard A. (2018). Futurism in nursing: Technology, robotics and the fundamentals of care. J. Clin. Nurs..

[5] Grinberg K., Sela Y. (2022). Perception of the image of the nursing profession and its relationship with quality of care. BMC Nurs..

[6] Liu N.Y., Hsu W.Y., Hung C.A., Wu P.L., Pai H.C. (2019). The effect of gender role orientation on student nurses’ caring behaviour and critical thinking. Int. J. Nurs. Stud..

[7] American Nurses Association (ANA) (2021). Nursing Scope & Standards of Practice.

[8] Brandão S., Peres M., Aparibense P.G., Lopes R., Santos J., Brandão M. (2020). Evidence of nursing patterns of knowing communicated by the brazilian press before Florence Nightingale’s model. Rev. Bras. Enferm..

[9] Hoseini T., Varasteh S., Esmaeili M. (2024). Explain the professional identity of nursing during COVID-19 pandemic. Nurs. Open.

[10] Smith C., Horne C. (2024). Educational and professional experiences of men in nursing: An interpretive description study to guide change and foster inclusive environments for men in nursing. J. Prof. Nurs..

[11] Smith C., Lane S., Brackney D., Horne C.E. (2020). Role expectations and workplace relations experienced by men in nursing: A qualitative study through an interpretive description lens. J. Adv. Nurs..

[12] Barros A., Menegaz J., Santos J., Polaro S., Trindade L., Meschial W. (2023). Nursing care management concepts: Scoping review. Rev. Bras. Enferm..

[13] Instituto Nacional de Estasítica. https://www.ine.es/jaxi/Datos.htm?tpx=49002.

[14] Ahmadi F., Shaker H., Eterafi M., Kamran A. (2023). Exploring nursing students’ perceptions from nursing role function (SP-NRF) during the COVID-19 pandemic in Ardabil Province: A cross-sectional study from Iran. BMC Nurs..

[15] Dost A., Bahçecik A.N. (2022). Determination of professional image perceptions of nursing students. J. Educ. Res. Nurs..

[16] Sillero A., Gil M., Marques-Sulem E., Ayuso R. (2023). Motivations and expectations of generation Z nursing students: A post-pandemic career choice qualitative analysis. J. Prof. Nurs..

[17] Rodríguez-González R., Martínez-Santos A., De La Fuente N., López-Pérez M., Fernandez-De-La-Iglesia J. (2023). Identifying engagement and associated factors in nursing students: An exploratory study. J. Prof. Nurs..

[18] Rubinstein D., Rubinstein D., Tabak N. (2013). Professional identity and image as factors that affect the profession. Contemporary Nursing Ethics.

[19] Nursing and Midwifery Council (2018). The Code. https://www.nmc.org.uk/standards/code/.

[20] Abdelrahman S. (2018). Relationship among public nursing image, self-image, and self-esteem of nurses. Nurs. Health Sci..

[21] López-Verdugo M., Ponce-Blandón J.A., López-Narbona F.J., Romero-Castillo R., Guerra-Martín M.D. (2021). Social image of nursing. An integrative review about a yet unknown profession. Nurs. Rep..

[22] Maliheh N.M., Ashraf A., Hamid H., Taghi S.M., Fatemeh H.N. (2021). The Public Nursing Image as Perceived by Nurses and Citizens: A Questionnaire Survey. Int. J. Caring Sci..

[23] Kristoffersen M. (2021). Does professional identity play a critical role in the choice to remain in the nursing profession?. Nurs. Open.

[24] Zhou Y., Weng L., Wang M., Huang G. (2024). Male nursing students’ experiences of their clinical internships: A qualitative study. Heliyon.

[25] Ulrich B. (2023). What’s your professional identity as a nurse?. Nephr. Nurs. J..

[26] Cajachagua-Castro M., Roque-Guerra E., Conque-Machaca N., Mamani-Contreras R., Chavez-Sosa J. (2022). Cuidado invisible e Imagen social de la enfermera comunitaria. ENE.

[27] Atashzadeh-Shoorideh F., Monjazabi F., Fathollahzadeh E., Parastoo O. (2021). The obstacles to nurses being present with patients. Nurs. Open.

[28] Chen X., Du Y., Shen Z., Qin W., Zhang Y. (2024). How the public perceives the “good nurse” in China: A content analysis of national newspapers. J. Nurs. Scholarsh..

[29] Wałowska K., Domaradzki J. (2023). Superheroes or Super Spreaders? The Impact of the COVID-19 Pandemic on Social Attitudes towards Nurses: A Qualitative Study from Poland. Int. J. Environ. Res. Public Health.

[30] Siebens K., De Casterlé B.D., Abraham I., Dierckx K., Braes T., Darras E., Dubois Y., Milisen K., BELIMAGE Group (2006). The professional self-image of nurses in Belgian hospitals: A cross-sectional questionnaire survey. Int. J. Nurs. Stud..

[31] Porter R.T., Porter M.J. (1991). Career development: Our professional responsibility. J. Prof. Nurs..

[32] Arthur D. (1995). Measurement of the professional self-concept of nurses: Developing a measurement instrument. Nurse Educ. Today.

[33] Angel E., Craven R., Denson N. (2012). The nurses self-concept instrument (NSCI): Assessment of psychometric properties for Australian domestic and international student nurses. Int. J. Nurs. Stud..

[34] Cowin L. (2001). Measuring Nurses’ Self-Concept. West. J. Nurs. Res..

[35] Dagenais F., Meleis A.I. (1982). Professionalism, work ethic, and empathy in nursing: The nurse self-description form. West. J. Nurs. Res..

[36] Godsey J., Hayes T., Schertzer C., Kallmeyer R., Mukherjee A., Weber J. (2018). Development and testing of three unique scales measuring the brand image of nursing. Int. J. Pharm. Healthc. Mark..

[37] Özsoy S.A. (2000). Determination of nursing image in the community. J. Ege Univ. Fac. Nurs..

[38] Toth J.C., Dobratz M.A., Boni M.S. (1998). Attitude toward nursing of students earning a second degree and traditional baccalaureate students: Are they different?. Nurs. Outlook.

[39] Hoskins L.M. (1983). View of Nursing Questionnaire [Mimeograph].

[40] Cukljek S., Juresa V., Babic J. (2017). The cross-cultural (transcultural) adaptation and validation of the nursing image questionnaire. Nurse Educ. Today.

[41] Hung C.-A., Wu P.-L., Liu N.-Y., Hsu W.-Y., Lee B.-O., Pai H.-C. (2019). The effect of gender-friendliness barriers on perceived image in nursing and caring behaviour among male nursing students. J. Clin. Nurs..

[42] Wang Q., Cao X., Du T. (2022). First-year nursing students’ initial contact with the clinical learning environment: Impacts on their empathy levels and perceptions of professional identity. BMC Nurs..

[43] Liebig D., Embree J.L., Lough C. (2024). Values and Ethics Domain for Professional Identity in Nursing. J. Contin. Educ. Nurs..

[44] Ding X., Wang L., Sun J., Li D., Zheng B., He S., Latour J.M. (2019). Effectiveness of empathy clinical education for children’s nursing students: A quasi-experimental study. Nurse Educ. Today.

[45] Çınar Ş., Demir Y. (2010). Nursing Image in Society: A Scale Development Study. Anat. J. Nurs. Health Sci..

[46] Hojat M., Mangione S., Kane G., Gonnella J.S. (2005). Relationships between scores of the Jefferson Scale of Physician Empathy (JSPE) and the Interpersonal Reactivity Index (IRI). Med. Teach..

[47] Weis D., Schank M.J. (2009). Development and psychometric evaluation of the Nurses Professional Values Scale Revised. J. Nurs. Meas..

[48] Leal-Costa C., Tirado S., Ramos-Morcillo A.J., Ruzafa-Martínez M., Díaz J.L., Van-der Hofstadt C.J. (2020). Communication Skills and Professional Practice: Does It Increase Self-Efficacy in Nurses?. Front. Psychol..

[49] Sousa V.D., Rojjanasrirat W. (2011). Translation, adaptation and validation of instruments or scales for use in cross-cultural health care research: A clear and user-friendly guideline. J. Eval. Clin. Pract..

[50] Kirchherr J., Charles K. (2018). Enhancing the sample diversity of snowball samples: Recommendations from a research project on anti-dam movements in Southeast Asia. PLoS ONE.

[51] World Medical Association (2013). World Medical Association Declaration of Helsinki: Ethical principles for medical research involving human subjects. JAMA.

[52] Bentler P.M., Bonett D.G. (1980). Significance tests and goodness of fit in the analysis of covariance structures. Acad. Psychol. Bull..

[53] Jöreskog K.G., Sörbom D. (1993). LISREL 8: User’s Guide.

[54] Hu L., Bentler P.M. (1999). Cutoff criteria for fi t indexes in covariance structure analysis: Conventional criteria versus new alternatives. Struct. Equ. Model. Multidiscip. J..

[55] Hair J.F., Anderson R.E., Tatham R.L., Black W.C. (1999). Análisis Multivariante.

[56] James L.R., Mulaik S.A., Brett J.M. (1982). Causal Analysis: Models, Assumptions and Data.

[57] Byrne B.M. (2001). Structural Equation Modeling with AMOS Basic Concepts, Applications, and Programming.

[58] Arbuckle J.L. (2006). Amos 7.0 User’s Guide.

[59] West S.G., Finch J.F., Curran P.J., Hoyle R.H. (1995). Structural equation models with non-normal variables. Structural Equation Modeling: Concepts, Issues and Applications.

[60] Bollen K.A., Stine R.A., Bollen K.A., Long J.S. (1993). Bootstrapping goodness-of-fit measures in structural equation models. Testing Structural Equation Models.

[61] Cohen J. (1992). A Power Primer. Psychol. Bull..

[62] Zhou L., Sukpasjaroen K., Cai E., Moonsri K., Imsiri P., Chankoson T. (2023). The psychometric properties of nursing image measurement instruments: A systematic review. Nurs. Open.

[63] Solera-Gómez S., Sancho-Cantus D., Privado J., Cunha-Pérez C., Benedito-Monleón A., Llinares-Insa L.-I. (In review) Adaptation and validation of the Nursing Image Scale in a Spanish population. Int. Nurs. Rev..

[64] Fernández-Feito A., Palmeiro-Longo M., Hoyuelos S., García-Díaz V. (2019). How work setting and job experience affect professional nurses’ values. Nurs. Ethics.

[65] Blanco J.M., Blanco A., Caballero F., Hawkins M., Fernández T., Lledó L., López A., Piñas A., Vara E., Monge D. (2022). Medical empathy in medical students in Madrid: A proposal for empathy level cut-off points for Spain. PLoS ONE.

[66] Montilva M., Garcia M., Torres A., Puertas M., Zapata E. (2015). Empatía según la escala de Jefferson en estudiantes de Medicina y Enfermería en Venezuela. Investig. Educ. Méd..

